# Proliferative and Regenerative Effect of Acetonic Extract of *Feijoa sellowiana* on Stem Cells

**DOI:** 10.29252/wjps.9.3.313

**Published:** 2020-09

**Authors:** Hosein Rasekh, Mehdi Hoseini Farahi, Davood Mehrabani, Seyed Jalil Massoumi, Mani Ramzi, Jason P. Acker

**Affiliations:** 1 Department of Horticultural Sciences, Yasooj Branch, Islamic Azad University, Yasooj, Iran;; 2 Stem Cell Technology Research Center, Department of Pathology, Shiraz University of Medical Sciences, Shiraz, Iran;; 3 Burn and Wound Healing Research Center, Department of Pathology, Shiraz University of Medical Sciences, Shiraz, Iran;; 4 Comparative and Experimental Medicine Center, Shiraz University of Medical Sciences, Shiraz, Iran;; 5 Department of Pathology, University of Alberta, Edmonton, AB, Canada;; 6 Nutrition and Food Sciences Research Center, Department of Nutrition, School of Nutrition and Food Sciences, Shiraz University of Medical Sciences, Shiraz, Iran;; 7 Hematology Research Center, Shiraz University of Medical Sciences, Shiraz, Iran;; 8 Centre for Innovation, Canadian Blood Services, Edmonton, AB, Canada

**Keywords:** Feijoa sellowiana, Bone marrow stem cells, Growth kinetics, Apoptosis

## Abstract

**BACKGROUND:**

Feijoa is widely used in medicine due to their anti-inflammatory, antioxidant, antimicrobial and antitumor properties. The current investigation studied the proliferative and regenerative effect of acetonic extract of *Feijoa sellowiana* on stem cells.

**METHODS:**

Acetone extract of Feijoa was prepared using percolator and rotary machines. Human bone marrow stem cells (hBMSCs) were used as experimental *in vitro* model and characterized morphologically, by flowcytometry, and differentiation properties. The toxicity of the extract on hBMSCs was determined by MTT assay. The viability and growth kinetics of hBMSCs treated to Feijoa was determined. Real time PCR was used for changes in expression of proliferative and apoptotic genes on day 7^th^.

**RESULTS:**

MTT assay demondtrated that Feijoa at doses less than 200 ng/ml did not show any cytotoxic effect on hBMSCs and increased the cell proliferation until day 3^rd^ followed by a non-significant slow decreasing trend until day 7^th^. Population doubling time (PDT) showed a decline until day 3^rd^ followed by an increase until day 7^th^. A significant rise in expression of Bax and decline in Bcl-2 expression were noted on day 7^th^.

**CONCLUSION:**

The modulatory activity of Feijoa may be responsible for its increasing effect on cell proliferation till day 3^rd^. Therefore, when faster proliferation during a shorter time period is targeted, Feijoa can be safely added to the culture media in the first three days.

## INTRODUCTION

Feijoa (*Feijoa sellowiana* (O. Berg) Burret) is an evergreen tree belonging to Myrtaceae family, with gray branches, elliptical buds, white and red flowers, and sweet-smelling leaves and is indigenous to western highlands of Paraguay, southern Brazil, Uruguay and northern Argentina.^1^^-^^6^ This plant entered the Mediterranean region in the late nineteenth century and also entered into Islamic Republic of Iran in 1973 through the Republic of Azerbaijan. This shrub is just grown on the northern strip of Iran.^7^ Feijoa has several biological features such as antibacterial,^8^ analgesic and anti-inflammatory,^9^^-^^11^ antioxidant and anticancer^12^^,^^13^ effects. 

The leaves of Feijoa are waste materials without any utilization and little research has been done on the leaves which have phenolic compounds.^14^ Compounds such as a-tocopherol, flavone, stigmasterol, b-carotene, some unreported longchain esters of tyrosol and a novel galactolipid have been extracted from the leaves of Feijoa.^15^^,^^16^ Moreover, antioxidant activity of the aqueous extract of Feijoa has previously been demonstrated.^17^

Investigation on human myeloid leukemia cells showed that flavonoids in Feijoa induce apoptosis through caspase activation, overexpression of p16, p21 and tumor necrosis factor-related apoptosis-inducing ligand (TRAIL), increase in histone and non-histone acetylation and inhibition in histone deacetylase (HDAC) pathways.^18^ The antioxidant activity of methanolic extract of Feijoa was illustrated in mouse liver via 3,4-methylenedioxymethamphetamine (MDMA, or ecstasy) denoting to the medicinal property of Feijoa. MDMA is a ring-substituted amphetamine derivative that was synthesized in 1912 by Merck chemical company and has attracted a great deal of attention in recent years. It has widespread abuse as a recreational drug by the young generation.^19^ This study was undertaken to evaluate the proliferative and regenerative effect of acetonic extract of *Feijoa sellowiana *on human bone marrow derived stem cells (hBMSCs).

## MATERIALS AND METHODS

Feijoa (*Feijoa sellowiana *[(O. Berg) Burret] leaves were collected from Ramsar Research Center, northern Iran in spring of 2017. To remove the epiphytic hosts, which are normally present on the surface, the leaves were treated with 0.8% Triton X-100. Distilled water was used for extensive washing and then the leaves were dried on filter papers in a dark and dry place for two weeks and were later changed into powder. Totally, 100 g of the powder was placed in a percolator machine for 72 hours in 1000 mL of acetone (Merck, Germany), while the acetone solvent was rotated at 45°C and 50 rpm in a rotary machine (IKA, Germany) to be completely evaporated for the solvent.^20^

The study was approved by Islamic Azad University Ethics Committee, Yasooj Branch, Yasooj, Iran (30/99-1111/519). Bone marrow (BM) samples were provided from the Bone Marrow Transplantation Center of Nemazi Hospital, affiliated to Shiraz University of Medical Sciences, Shiraz, Iran. A written signed consent form was provided from the subjects by Hematology Research Center, Shiraz University of Medical Sciences, Shiraz, Iran. BM was diluted with an equal volume of Dulbecco’s Modified Eagle Medium (DMEM; Biovet, Bulgaria) and was carefully layered over an equal volume of Ficoll-Paque product (without intermixing, GE Healthcare Life Sciences, UK) as described before.^21^

It was centrifuged at 1500 rpm and 20°C for 30 min to separate a second layer. The interface between the plasma and the Ficoll-Paque layer consisted of mononuclear cells (MNCs) were seeded in 75 cm flasks containing 88% alpha minimal essential medium (αMEM; Biovet, Bulgaria), 10% fetal bovine serum (FBS; Biovet, Bulgaria), 1% penicillin and streptomycin (Biovet, Bulgaria), and 1% L-glutamine (Sigma, USA). hBMSCs were transferred in 5% CO_2_ incubator at 37°C with saturated humidity (Memmert, Germany). The medium was changed every 3 days until 80% confluence and sub-cultured using 0.25% trypsin (Gibco, USA) till passage 3^rd^. 

The adhered cells were assessed morphologically to be spindle shape. *In vitro* differentiation to osteogenic lineage was assessed by seeding 5×10^4^ hBMSCs in a 12-well plate containing osteogenic medium of DMEM, 10% FBS, 200 μM L-ascorbic acid, 10 mM glycerol phosphate and 100 nM dexamethasone, while the media was changed every three days until 3 weeks. Alizarin red (Sigma-Aldrich, USA) staining that bound to calcium mineralized deposits revealing a red color when osteogenic differentiation was induced.

*In vitro* differentiation to adipogenic lineage was investigated by seeding 5×10^4^ hBMSCs in a 12-well plate containing adipogenic medium of DMEM, 10% FBS, 1 μmol/L dexamethasone, 200 μg/mL insulin, 0.5 mmol/L isobutylmethylxanthine, and 60 μmol/L indomethacin (all from Sigma-Aldrich, USA) for 21 days, while the medium was replaced every 3 days. Then, the cells were stained with Oil red O (Sigma-Aldrich, USA) to evaluate presence of red color droplets, when adipogenic differentiation was induced. Flowcytometry of hBMSCs was conducted for expression of mesenchymal markers (CD73 and CD90) and hematopoietic markers (CD34, and CD45) (Dako, Denmark) in passage 3^rd^.

**Fig. 1:**
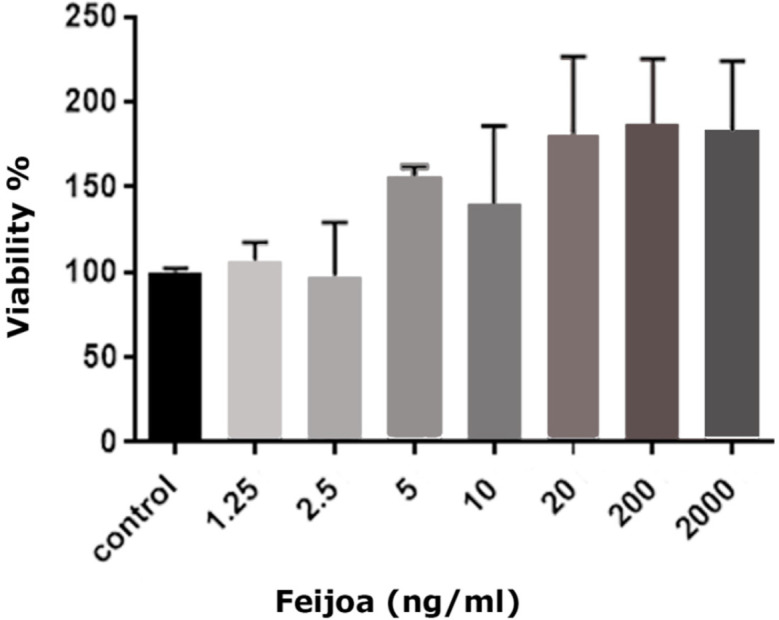
MTT assay of different concentrations of Feijoa after 7 days regarding the cell viability

MTT assay was done to determine the probable toxicity of Feijoa for hBMSCs. Totally, 1×10^4^ hBMSCs were seeded in a 96-well plate (Invitrogen, USA). After 24 hours, the medium was changed and different doses of Feijoa (1.25, 2.5, 5, 10, 20, 200 and 2000 ng/mL) were added and transferred to 5% CO2 incubator for one day at 37°C and saturated humidity. Twenty micro-liter of MTT was added for 4 hours for each 200 μL of the culture medium. It was later centrifuged for 5 minutes, the supernatant was removed and 200 μL of DMSO (Sigma-Aldrich, USA) was added to the remaining. Optical absorption at 570 nm was read by an ELISA plate reader (Polarstar Omega-BMG Lab Tech, Germany). Based on the highest percentage of cell viability that 200 μL/mL of Feijoa induced in hBMSCs until 7 days, this concentration was selected as optimum dose in all experiments (Figure 1). 

hBMSCs from passage 3^rd^ were seeded in 24-well culture plates at a density of 4×10^4^ cells per well and transferred in a 5% CO_2_ incubator at 37°C and saturated humidity. After 3 days, the culture media was changed and Feijoa at different time intervals was added. Acetone was used as a control. To evaluate cell viability, 0.4% trypan blue solution (Biowest, France) was added to the cell suspension and counted each day using a Neubauer hemocytometer slide and a phase contrast microscope until 7 days. The cell viability was calculated at optical density of 570 nm on a microplate reader (Floustar Omega, BMG LabTech, Germany) (n=4) using the following formula, while Ac and Ab were considered as the absorbance in the control and blank wells. %Survival rate=A sample–(Ab×Ac–Ab)×100.^22^

The population doubling time (PDT) was measured and the growth curve was plotted using the following the formula of PDT=T ln2=ln(Xe/Xb); while T is the incubation time in hours, Xb is the cell number at the beginning of the incubation time, and Xe is considered as the cell number at the end of the incubation time. The mean number of cells at each time point was plotted by GraphPad Prism (GraphPad software Inc., San Diego, CA, USA). Cells were cryopreserved in 10% (V/V) dimethyl sulphoxide (DMSO; MP Bio USA), 50% (V/V) fetal bovine serum and 40% DMEM, at a density of 1×10^6^ cells/mL until use.^22^

Total cellular RNA was isolated from hBMSCs using RNA extraction kit (CinnaGen, Iran). Applying a nanodrop™ spectrophotometer (Nanodrop; Thermo Fisher Scientific, USA), the quantity and quality of RNA were assessed by determining the ratio of optical density at 260/280 nm. They were stored at 80˚C until cDNA synthesis. The cDNA was synthesized by 1,000 ng total RNA in a first strand cDNA synthesis reaction using RevertAid™ First Strand cDNA Synthesis kit (Thermo Fisher Scientific, USA).^22^

Quantitative real time PCR (qPCR) was conducted by ABI Biosystems StepOne and the RealQ Plus 2x Master Mix Green (Ampliqon A/S, Odense, Denmark). In each reaction, 200 nM of each primer (Table 1) was added to target the specific sequence. Specific primers targeting Bax and Bcl-2 were prepared (Table 1), while the TBP housekeeping gene was as internal control for qPCR reactions. The qPCR condition was established for 10 minutes at 94˚C followed by 40 cycles of 15 sec at 94˚C, 60 sec at 58˚C and final extension of 7 minutes at 72˚C. The amplification signals of different samples were normalized to TBP cycle threshold (Ct), and then 2 ΔΔCq methods were applied to compare mRNA level of activated vs. the control, which were shown as fold change in data analysis. The data was analyzed by independent samples t test, using GraphPad Prism 6 (GraphPad Software, Inc., La Jolla, CA, USA). The P<0.05 was considered statistically significant.

**Table 1:** The specific primer sequences of the targeted genes

**Genes**	**Primer sequences**	**Size (bp)**
BAX	Forward: 5’- GCCCTTTTGCTTCAGGGTTTCA -3’	108
	Reverse: 5’- CAGCTTCTTGGTGGACGCAT -3’	
Bcl-2	Forward: 5’- ACGAGTGGGATGCGGGAGATGTG-3’	245
	Reverse: 5’- GCGGTAGCGGCGGGAGAAGTC-3’	
TBP	Forward: 5’- GGATAAGAGAGCCACGAACCAC-3’	139
	Reverse: 5’- TTAGCTGGAAAACCCAACTTCTG-3’	

bp, base pair

## RESULTS

**Fig. 2:**
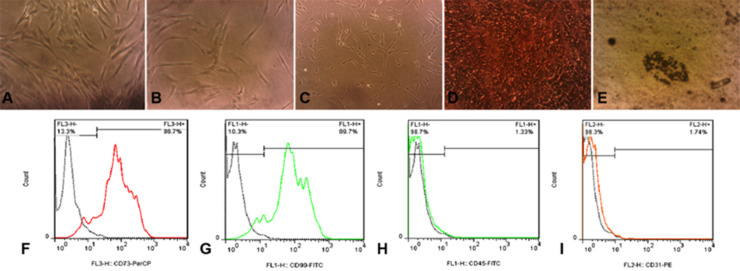
hBMSCs in Passage 1 (A), Passage 2 (B), and Passage 3 (C) (100×). Osteogenic induction (D), and adipogenic differentiation (E). Flowcytometry being positive for mesenchymal markers of CD73 (F) and CD90 (G) and negative for hematopoietic markers of CD34 (H) and CD45 (I).

hBMSCs were all adhered to the culture flasks. These cells were spindle shape in different passages (Figure 2). Three weeks later, these cells in osteogenic media showed presence of calcium deposits confirmed by Alizarin red staining in red color (Figure 2). Regarding adipogenic differentiation, hBMSCs were positive for red oil droplets in adipogenic media (Figure 2). The cells were also positive for expression of mesenchymal markers of CD73 and CD90, and negative for hematopoietic markers of CD34 and CD45 (Figure 2). 

**Fig. 3:**
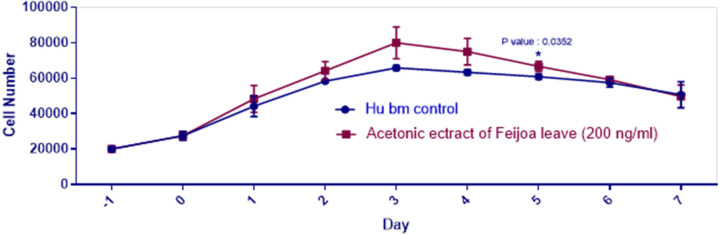
The growth curve of hBMSCs treated with 200 ng/mL of acetonic extract of Feijoa leave during seven days in comparison to the control group (P<0.05). Hu bm: Human bone marrow

**Fig. 4:**
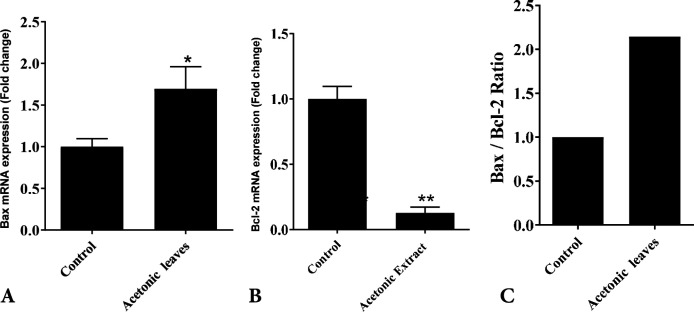
The expression of Bax (P<0.05) (A) and Bcl-2 genes (P<0.01) (B) and the ratio of Bax\Bcl-2 (C) genes for control and hBMSCs treated with 200 ng/mL of acetonic extract of Feijoa leave on day 7^th^.

MTT assay showed that acetonic extract of Feijoa leaves had no cytotoxic effects on hBMSCs at doses ≤200 ng/mL (Figure 2). PDT over seven days in control and hBMSCs groups treated with 200 ng/mL of acetonic extract of Feijoa was presented in Table 2. PDT showed a decline until day 3^rd^ followed by an increase until day 7^th^. The growth curve was plotted based on the average number of cells treated with 200 ng/mL of acetonic extract of Feijoa during a seven days period. An increasing trend in cell proliferation till day 3 was noted, while proliferation demonstrated a further decrease when compared to the control group (Figure 3). Real time PCR for expression of Bax and Bcl-2 genes of the cells treated with 200 ng/mL of acetonic extract of Feijoa exhibited an increase in expression of Bax gene, and a decrease in expression of Bcl-2 gene (P≤0.05) on day 7^th^ (Figure 4). 

**Table 2:** PDT of control and treated cells to 200 ng/mL of acetonic extract of Feijoa leave

**Variable**	**Population doubling time (n)**	
**Day**	**Control **	**Feijoa (200 ng/mL)**
0	0	0
1	21	17
2	31	29
3	42	39
4	58	54
5	75	71
6	94	92
7	125	113

## DISCUSSION

In our study identical to other studies, hBMSCs had mesenchymal properties confirmed morphologically, by osteogenic and adipogenic induction and by flowcytometry.^23^ Regarding growth kinetics of these cells, we noted an increasing trend until day 3^rd^, and then a decrease until day 7^th^ when exposed to the acetonic extract of Feijoa. It was shown that, when Caco-2 and HT-29 cells were exposed to *Feijoa sellowiana* for 24 hours, it demonstrated anti-inflammatory effects^24^ and prevented lipid peroxidation in intestinal epithelial cells.^25^^,^^26^ These findings may explain the increasing trend noted in proliferation of hBMSCs during the first 3 days of our study. Feijoa can also improve the lactase and sucrase-isomaltase activities and inhibit cell proliferation too. It has anticancer activities.^27^ These activities may be responsible for the decreasing effect of Feijoa on proliferation of hBMSCs and can explain the growth kinetics in our study. 

Bax and Bcl-2 apoptotic genes have been of tremendous interest to clinicians who study cancer therapy. There are two classes of Bcl-2 proteins including pro-apoptotic proteins (Bax, Bad, Bid, Bik) and anti-apoptotic proteins (Bcl-2, Bcl-XL, Bcl-W), while anti-apoptotic proteins are responsible for apoptosis through delaying the mitochondrial release of cytochrome-c, and that the proapoptotic proteins activate such releases.^28^ Bax as a pro-apoptotic protein was shown to trigger apoptosis by increasing the opening of the mitochondrial voltage-dependent anion channels, which induce the loss in its membrane potential. The protein Bcl-2 is a key inhibitor of apoptosis, and its aberrant expression was demonstrated in a wide range of solid tumors.^29^ The ratio of Bax to Bcl-2 denotes to the susceptibility of a cell to apoptosis too.^30^ Our findings revealed that Feijoa significantly increased the expression of Bax gene, while decreased Bcl-2 expression, suggesting that apoptotic mechanisms of Feijoa are responsible for up regulation of Bax expression.^30^

These results are consistent with other studies reporting that Bax and Bcl-2 play an important role in apoptosis, growth kinetics and cell differentiation. Our results resemble the ﬁndings of cytotoxic potential effects of *E. billardieri* on MCF-7, A549, HepG-2 and HT-29 cell lines.^31^^-^^33^

## CONCLUSION

The modulatory activity of Feijoa may be responsible for its increasing effect on cell proliferation till day 3^rd^. Therefore, when faster proliferation during a shorter time period is targeted, Feijoa can be safely added to the culture media in the first three days. This study also revealed that acetonic leave extract of Feijoa significantly induced apoptosis on day 7^th^ by increasing expression of Bax and decreasing of Bcl-2 genes. However, more molecular studies should be undertaken to achieve a definite conclusion to determine the probable apoptotic pathways of the cytotoxic activity of Feijoa on day 7^th^. 
